# *Aeromonas hydrophila* utilizes TLR4 topology for synchronous activation of MyD88 and TRIF to orchestrate anti-inflammatory responses in zebrafish

**DOI:** 10.1038/cddiscovery.2017.67

**Published:** 2017-10-02

**Authors:** Nidhi Srivastava, Asha Shelly, Manmohan Kumar, Archana Pant, Bhabatosh Das, Tanmay Majumdar, Shibnath Mazumder

**Affiliations:** 1 Immunobiology Laboratory, Department of Zoology, University of Delhi, Delhi, India; 2 Translational Health Science and Technology Institute, NCR Biotech Science Cluster, Faridabad, India; 3 School of Life Sciences, Manipal University, Manipal, Karnataka, India

## Abstract

Toll-like receptor 4 (TLR4) plays a critical role in host immunity against Gram-negative bacteria. It transduces signals through two distinct TIR-domain-containing adaptors, MyD88 and TRIF, which function at the plasma membrane and endosomes, respectively. Using zebrafish *Aeromonas hydrophila* infection model, we demonstrate that synchronization of MyD88 and TRIF dependent pathways is critical for determining the fate of infection. Zebrafish were infected with *A. hydrophila,* and bacterial recovery studies suggested its effective persistence inside the host. Histopathological assessment elucidates that *A. hydrophila* did not provoke inflammatory responses in the spleen. Immunofluorescence revealed the presence of TLR4-bound *A. hydrophila* on the plasma membrane at 3 h post-infection (p.i.), and inside endosomes 1 day p.i. Quantitative PCR studies suggest that TLR4 activates the downstream pathway of MyD88–IRAK4 axis at early stages followed by a shift to TRIF–TRAF6 axis at late stages of infection coupled with fold increase in NF*κ*B. Our results implicated the involvement of p110*δ* isoform of PI(3)Kinase in this transition. Coupled to this, we noted that the TLR4–TRIF–NF*κ*B axis prompted burgeoned secretion of anti-inflammatory cytokines. We observed that *A. hydrophila* inhibits endosome maturation and escapes to cytoplasm. Significant downregulation of cytosolic-NLR receptors further suggested that* A. hydrophila* represses pro-inflammatory responses in cytosol aiding its persistence. Our findings suggest a novel role of ‘TLR4 topology’ in *A. hydrophila*-induced pathogenesis. We propose that *A. hydrophila* manipulates translocation of TLR4 and migrates to endosome, where it triggers TRIF-dependent anti-inflammatory responses, interferes with endosomal maturation and escapes to cytosol. Inside the cytosol, *A. hydrophila* avoids detection by suppressing NLRs, facilitating its survival and ensuing pathogenesis.

## Introduction

The sensing of microbes is mediated by innate pattern recognition receptors (PRRs), which include Toll-like receptors (TLRs).^1^ TLRs belong to type 1 transmembrane protein family and comprise an N-terminal extracellular domain that recognizes pathogen-associated molecular patterns, transmembrane domain and C-terminal or intracellular Toll/interleukin-1 receptor (TIR) domain which serves as a docking site for adaptor molecules important for downstream signaling.^2^ Among TLR family members, TLR4 was the first identified and recognized as an important innate immune receptor.^3,4^ Once TLR4 binds to its cognate ligand, lipopolysaccharide of Gram-negative bacteria, it activates two distinct signaling pathways – the MyD88-dependent and TRIF-dependent pathways. Signaling through these two pathways leads to expression of both pro- and anti-inflammatory cytokines and immunomodulatory molecules.^5^ MyD88-dependent signaling requires the sorting adaptor TIRAP/Mal at the plasma membrane, which helps in induction of NF*κ*B and mitogen-activated protein kinase (MAPK) pathways culminating in pro-inflammatory responses. On the other hand, the MyD88-independent or TRIF-dependent pathway is endosomal and initiated by the sorting adaptor TRAM. The TIRAP–MyD88 pathway transits sequentially into TRAM–TRIF signaling, though the mechanisms remain unknown. It was observed that p110*δ*, which is the main class I PI(3)K isoform, ‘licenses’ the internalization of TLR4 from the plasma membrane to endosome.^6^ The transition of TLR4 from an early-acting TIRAP–MyD88 associated plasma membrane complex into a late-acting endosomal TRAM–TRIF complex is often associated with switch from pro-inflammatory to anti-inflammatory responses.^5^

The presence of TLR4 is well reported in fish.^7–14^ However, the role of fish TLR4 as a receptor for LPS is contentious. Compared with mammals, fish can tolerate relatively higher concentrations of LPS.^15^ The lack of LBP, MD2 and CD14, essential for LPS binding to TLR4 in all fish genomes studied till date,^16^ clearly suggests the mechanism of LPS recognition in fish to be different from that of mammals.^7,16,17^ Zebrafish is known as a sentinel species to model human diseases^18^ as it has a fully mapped genome significantly homologous with that of humans.^19^ Zebrafish TLR4 has been cloned and characterized.^20,21^ It exists as two TLR4 paralogs – TLR4a and TLR4b^7^ – which show sequence difference in the extracellular protein domain.^17^ There are reports suggesting that zebrafish TLR4 fails to recognize LPS and negatively regulates MyD88–NF*κ*B axis.^7^

*Aeromonas hydrophila*, Gram-negative rod-shaped bacterium, shows wide host tropism. In fish, it causes aeromonad septicemia,^22,23^ though the pathogenesis is not well understood. The bacteria expresses diverse array of virulence factors which together with environmental cues manifest pathogenic effects in diverse hosts.^24,25^ In fish, spleen is an important immune organ that participates in mounting both innate and adaptive immune responses and helps in the clearance of microbial pathogens in fish.^26,27^ Although, there are several reports documenting the active involvement of several fish immune molecules during *A. hydrophila* infections,^28–32^ information regarding the role of TLR4 and the associated downstream signaling pathway in *A. hydrophila* pathogenesis still remains unexplored. To look into this we have used the zebrafish *A. hydrophila* infection model and report that the spatial distribution of TLR4 (plasma membrane vs endosomal) governs dichotomous TLR4 signaling, thereby influencing microbial pathogenesis.

## Results

### Clinical signs of *A. hydrophila* infection in zebrafish are dose dependent

**Figure 1:**
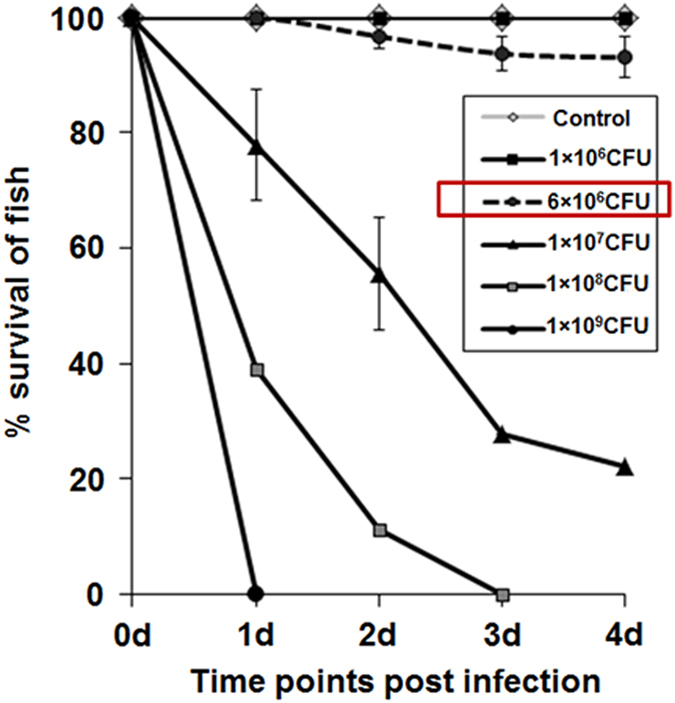
*A. hydrophila* induced dose-dependent mortality in zebrafish. Zebrafish were injected i.p. with different CFU of *A. hydrophila* and the mortality was recorded at indicated time points. Data represent the mean value of three independent experiments (*n*=10/experiment) and the error bars represent standard deviations. d, days.

At the onset we aimed to determine the LD50 dose of the *A. hydrophila* isolate in zebrafish. Zebrafish were infected with three different doses of live *A. hydrophila* and mortality along with phenotypic changes monitored. Infection with 1×10^9^ CFU led to 100% mortality within 1 day post-infection (p.i.) and the infected fish did not show any signs of disease manifestations. Infection with 1×10^7^ CFU led to 78% mortality within 4 days of infection (Figure 1) and the infected fish showed severe petechial hemorrhages on the abdomen as well as at the base of the fins. The fish stopped feeding, remained listless at the bottom of the tanks and rarely came to surface for gulping air. Infection with 1×10^6^ CFU failed to induce any phenotypic change and fish death (Figure 1). Based on the mortality results, the 4-day LD50 dose of *A. hydrophila* was calculated to be 8×10^6^ CFU. We selected 6×10^6^ CFU as the infection dose which caused 7% mortality over a period of 4 days (Figure 1) and the infected fish exhibited peritonitis and localized hemorrhagic lesions. Control and sham-injected fish did not show any mortality. All the dead fishes were homogenized, plated on *Aeromonas* selective medium plates and the presence of *A. hydrophila* colonies were noted (data not shown).

### Histological analysis elucidates absence of inflammation in zebrafish spleen

**Figure 2:**
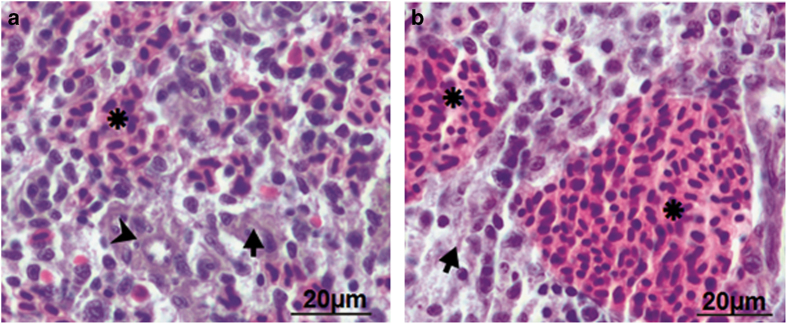
Histological analysis elucidated absence of inflammation in zebrafish spleen. Spleen was removed from control and infected zebrafish and the parenchyma of both was compared (×40). (**a**) Control zebrafish spleen comprises hematogenous red pulp (asterisk), lymphoid white pulp (arrow) and ellipsoid (arrow head). (**b**) *A. hydrophila-*infected fish spleen revealed significant expansion of the red pulp area.

The next step was studying *A. hydrophila*-induced histopathological alterations. For this we selected spleen, an important immune organ in fish. The parenchyma of spleen comprises of two major functional zones – the hematogenous red pulp and the lymphoid white pulp (Figure 2a). The red pulp houses the erythrocytes and thrombocytes while the white pulp possesses the lymphatic tissue. The presence of ellipsoids is often noted at the terminals of splenic arterioles (Figure 2a). In infected zebrafish, a significant expansion of the red pulp area was observed (Figure 2b). Other cellular alterations like hypertrophied cells, hyperplasia, lymphocyte infiltration and edematous swelling were not evident in the parenchyma of infected zebrafish spleen. These results suggest that *A. hydrophila* does not provoke pro-inflammatory changes in splenic parenchyma.

### TLR4 signaling is critical in *A. hydrophila* pathogenesis

**Figure 3:**
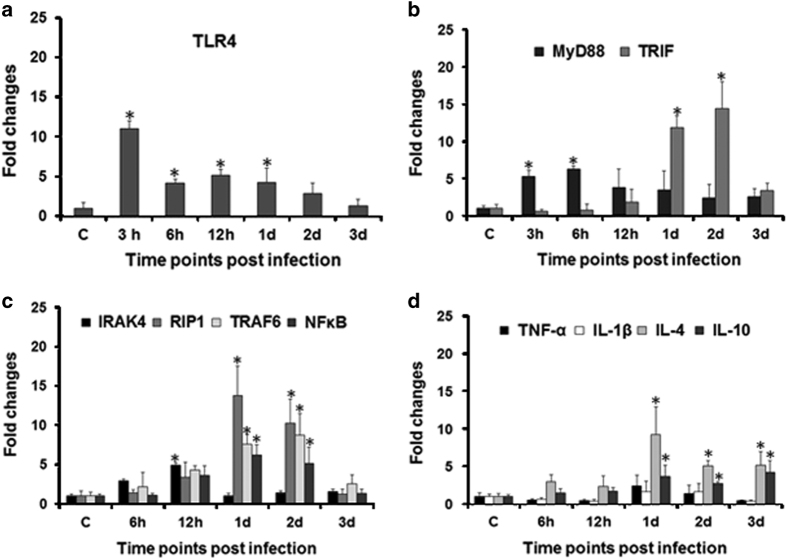
Expression studies of TLR4 signaling pathway-related genes. Spleen was removed from control and infected zebrafish at indicated time points, total RNA isolated, cDNA prepared which was used for qPCR analysis of (**a**) TLR4; (**b**) MyD88, TRIF; (**c**) IRAK4, RIP1, TRAF6, NF*κ*B; (**d**) TNF-*α*, IL-1*β*, IL-4, IL-10. Each bar represent the mean of three independent experiments (*n*=5/experiment) and the error bars represent the standard deviations. C, control; h, hours; d, days; and asterisks '∗' on bars indicate significant difference from control (*P*<0.05).

The role of fish TLR4 is well implicated in immunity to Gram-negative bacteria.^33^ We were interested to study TLR4 signaling in zebrafish spleen following *A. hydrophila* infection. To this end, zebrafish were infected with 6×10^6^ CFU of *A. hydrophila*, spleen removed and TLR4 expression monitored by qPCR at indicated time points. We observed maximum TLR4 expression at 3 h p.i., thereafter the levels though declined remained significantly high till 1 day p.i. (*P*<0.05) (Figure 3a). These results suggested the involvement of TLR4 in *A. hydrophila* pathogenesis in zebrafish.

### *A. hydrophila* triggers MyD88 and TRIF in a sequential manner

TLR4 signaling is mediated by two distinct adapter molecules, MyD88 and TRIF.^5,6^ In absence of prior information, we aimed to decipher the relative involvement of the two adapter molecules during the course of *A. hydrophila* pathogenesis in fish. It is evident from Figure 3b that significant MyD88 (*P*<0.05) expression occurred in spleen only at the earlier time points, that is, at 3 and 6 h p.i. On the contrary, significant expression of TRIF was noted at later time points with the maximum fold increase recorded in 2 days post-infected spleens (Figure 3b). Together, these results for the first time demonstrated differential TLR4 signaling in course of *A. hydrophila* pathogenesis wherein MyD88 pathway is involved at the early stages and TRIF pathway at later stages of infection.

### TLR4 signaling is divaricated in zebrafish spleen

Our next aim was to explore the downstream signaling cascade involved in *A. hydrophila* pathogenesis. We monitored the expression of IRAK4 (IL-1 receptor-associated kinase), TRAF6 and TRAF3 (TNF receptor-associated factors). It was observed that though IRAK4 expression started increasing from 6 h p.i. (*P*=0.063), significant increase was recorded at 12 h p.i. (*P*<0.05), thereafter it declined and reached basal levels at 1 day p.i. Our results suggested significant upregulation of RIP1 (*P*<0.05) and TRAF6 (*P*<0.05) at 1 and 2 days p.i. (Figure 3c). The expression of TRAF3 remained undetected throughout the experiment. We followed this by studying the fold change in NF*κ*B and it is evident from Figure 3c that its expression started increasing from 12 h p.i. (*P*=0.144) with a significant fold change recorded (*P*<0.05) in 1 and 2 days infected spleen. To this we infer that consequent to *A. hydrophila* infection two signaling pathways are initiated in zebrafish; at early stages TLR4–MyD88–IRAK4–TRAF6–NF*κ*B is functional and at the later stages signaling is through the TLR4–TRIF–RIP1–TRAF6–NF*κ*B axis.

### *A. hydrophila* pathogenesis induces anti-inflammatory response in zebrafish

The dichotomy in TLR4 downstream signaling pathways prompted us to study cytokine expression in course of *A. hydrophila* infection. Zebrafish were infected with *A. hydrophila* and the expression of key pro- and anti-inflammatory cytokines studied. We failed to detect the expression of pro-inflammatory cytokines IFN-*γ* and IL-12 at all the time points studied. This was followed by studying the kinetics of two other pro-inflammatory cytokines TNF-*α* and IL-1*β* and we did not observe any noticeable increase in their expression levels (Figure 3d). Contrastingly, the expression of IL-4 and IL-10 was found to be upregulated throughout the experiment with a significant fold increase (*P*<0.05) recorded from 1 day onwards in infected spleen. Collectively, these results suggested that *A. hydrophila* infection primarily induces anti-inflammatory responses in zebrafish.

### *A. hydrophila* persists inside zebrafish

**Figure 4:**
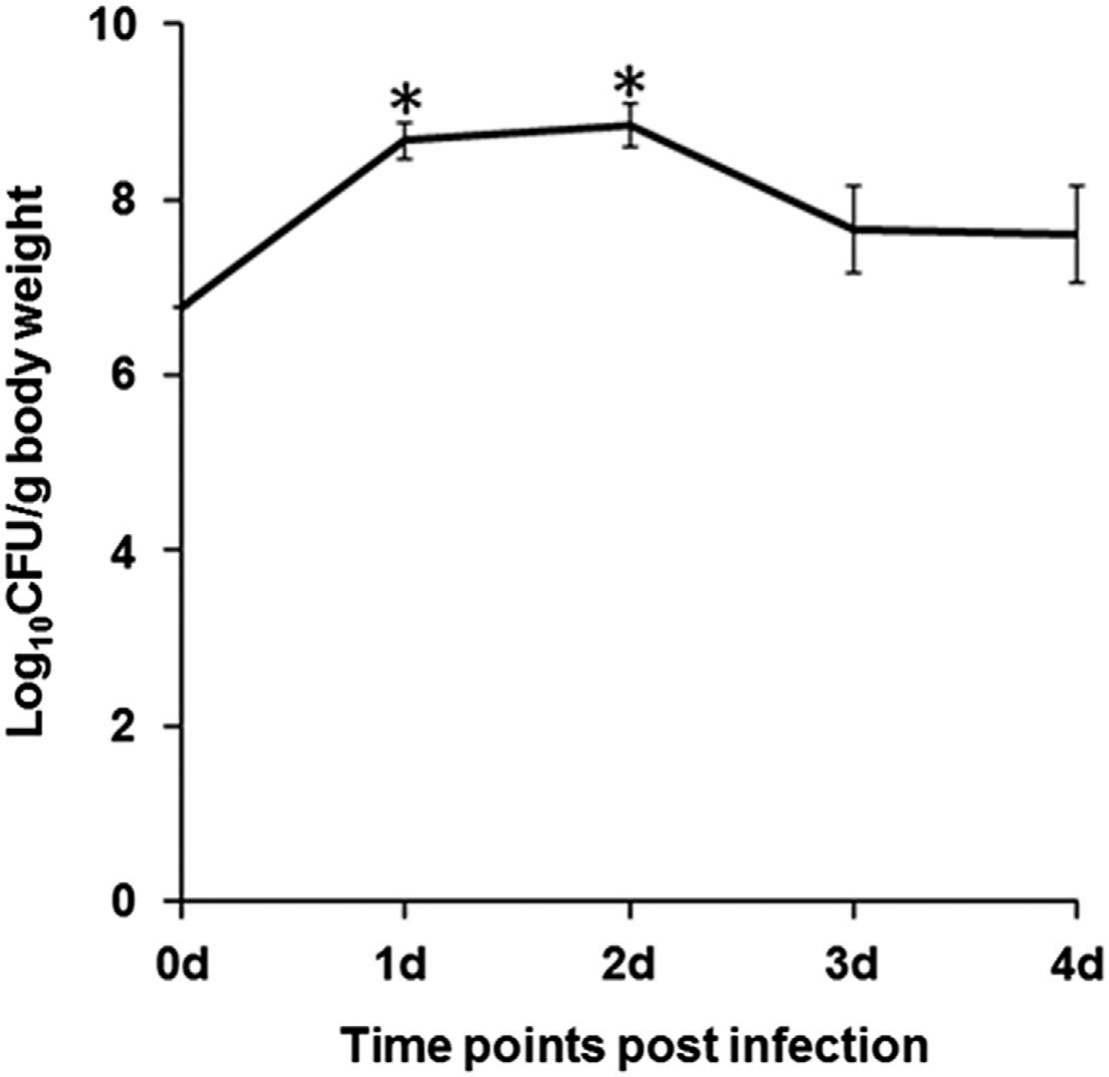
*A. hydrophila* persists in infected zebrafish. Zebrafish were infected with *A. hydrophila* (6×10^6^ CFU) and the bacterial load was studied in the whole fish by counting CFU at the indicated time points. Data represent mean value of three independent experiments (*n*=5/experiment) and the error bars represent standard deviations. d, days and asterisks '∗' indicate significant difference from CFU at the time of infection (*P*<0.05).

The next step was to correlate the anti-inflammatory response with bacterial persistence. Zebrafish were infected with live *A. hydrophila,* killed at indicated time points, head and fins removed, homogenized, plated on *Aeromonas* selective medium and bacterial load quantified by counting CFU. We observed significant increase in the bacterial load at 1 day p.i., which remained almost similar at 2 days. Thereafter, though it declined, the bacterial number never reached basal level (Figure 4). Control fish did not exhibit any bacterial colony at any time intervals.

### *A. hydrophila* gets endocytosed after being recognized at the membrane

**Figure 5:**
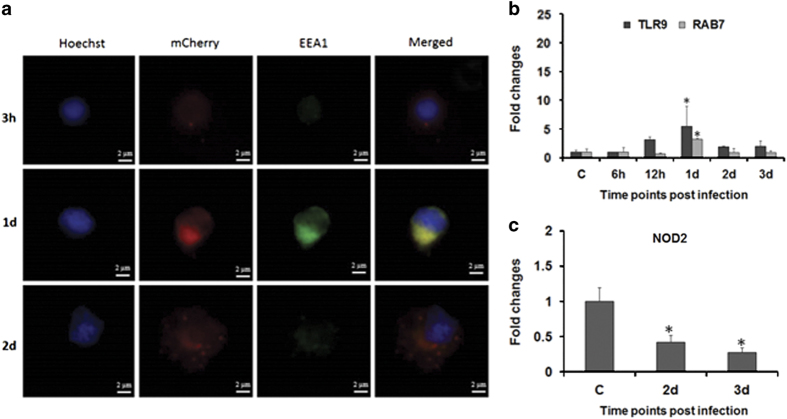
*A. hydrophila* trafficking in zebrafish. (**a**) Zebrafish were infected with mCherry-tagged *A. hydrophila*. Bacterial localization was revealed by immunofluorescence staining of zSPM using Hoechst 33342 and EEA1 at indicated time points (×100). (**b**, **c**) Expression of TLR9, RAB7 and NOD2 was determined from spleen of control and infected fishes. Each bar represent the mean of three independent experiments (*n*=5/experiment) and the error bars represent the standard deviations. C, control; d, days; h, hours; zSPM, zebrafish splenic macrophages; and asterisks '∗' on bars indicate significant difference from control (*P*<0.05).

The time kinetics of MyD88 as well as the TRIF-dependent pathway prompted us to explore the reason behind the differential activation of both the pathways. To study this, zebrafish were infected with mCherry-tagged *A. hydrophila* and bacterial movement inside splenic macrophages monitored at indicated time intervals using the early endosomal marker, EEA1 (green fluorescence). It is evident from our immunofluorescence studies that at the early stages of infection (3 h), the bacteria remain localized on the membrane of macrophages (red fluorescence) (Figure 5a). The yellow fluorescence (merged) observed in macrophages collected 1 day p.i. suggested the presence of bacteria in the early endosomes (Figure 5a). Macrophages obtained from 2 days post-infected spleen did not display yellow fluorescence but there were several red fluorescent dots in the cytoplasm (Figure 5a) indicating the presence of *A. hydrophila* in the cytoplasm of infected zebrafish splenic macrophages (zSPM).

To substantiate bacterial movement through endosomes, we monitored the expression of TLR9, an endosomal TLR. In corroboration to our immunofluorescence study we observed significant upregulation in TLR9 expression in 1 day infected spleen (*P*<0.05) followed by sudden decline from 2 days p.i. (*P*<0.05) (Figure 5b).

### *A. hydrophila* escapes from the early endosomes

The immunofluorescence results encouraged us to study *A. hydrophila* movement after egressing early endosomes. The expression of RAB7, a bonafide marker for the late endosomes, was checked, which revealed significant upregulation at 1 day p.i. (Figure 5b). Thereafter RAB7 expression declined reaching basal levels in 2 and 3 days post-infected spleen, suggesting limited transition from early to late endosome formation in *A. hydrophila*-infected macrophages. The presence of *A. hydrophila* in the cytoplasm prompted us to check the expression pattern of cytosolic sensor NOD2 at the later time points when the bacterium moved to the cytosol. We observed significant downregulation of NOD2 expression (*P*<0.05) in 2 and 3 days post-infected spleen (Figure 5c), suggesting its inhibition to be a survival strategy for *A. hydrophila* inside the macrophages.

### TLR4 internalization is supported by p110*δ* isoform of the kinase PI(3)K

**Figure 6:**
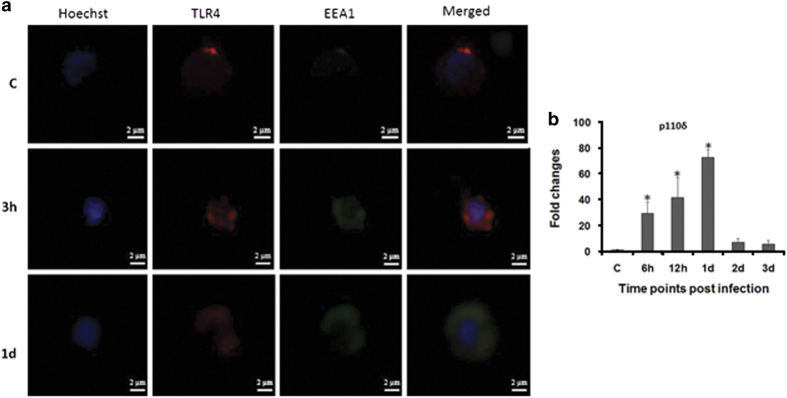
Subcellular distribution of TLR4. (**a**) zSPM were isolated from control and infected fish, stained with Hoechst 33342, TLR4 and EEA1 antibody for TLR4 internalization study (×100). (**b**) Expression of p110*δ* was determined from spleen of control and infected fishes. Each bar represent the mean of three independent experiments (*n*=5/experiment) and the error bars represent the standard deviations. C, control; d, days; h, hours; zSPM, zebrafish splenic macrophages and asterisks '∗' on bars indicate significant difference from control (*P*<0.05).

The dichotomy in TLR4 signaling prompted us to study its localization in infected zSPM. The zSPM were permeabilized and stained with TRITC-conjugated anti-TLR4 antibody and FITC-conjugated anti-EEA1 at indicated time points and observed under a fluorescence microscope. Red fluorescence observed in control zSPM and at the early stage of infection (3 h) advocates that initially TLR4s remain at the membrane (Figure 6a) but at 1 day p.i. the appearance of yellow fluorescence indicates that they get internalized into the endosome at this time point (Figure 6a). The internalization of TLR4 is mediated through p110*δ* isoform of the kinase PI(3)K.^6^ Our qPCR data support this statement as we noted maximum upregulation of p110*δ* mRNA expression in zSPM collected from 1 day post-infected spleen (*P*<0.05) followed by its decline from 2 days onwards in the infected spleen (Figure 6b).

## Discussion

*A. hydrophila*-induced pathogenesis in fish is not well understood. Though there are reports suggesting TLR4 imperative for innate recognition and regulating *A. hydrophila* pathogenesis in fish,^8,11,33–35^ the underlying mechanisms remain unknown. In mammals, the temporal distribution of TLR4 between plasma membrane and endosome facilitates its interaction with the adaptor molecules MyD88 and TRIF, respectively, dictating the outcome of innate immune signaling. We were interested to study how fish TLR4 co-ordinates the activation of the two distinct pathways ensuing *A. hydrophila* pathogenesis.

We observed maximum TLR4 expression at the earlier stages of infection, thereafter the levels though declined remained significant till 1 day p.i. in the spleen. The early expression of TLR4 correlates with its sentinel role as PRR during microbial infections. At early stages, plasma membrane-associated TLR4 engages MyD88 to trigger the production of pro-inflammatory cytokines.^5^ We noted significant expression of MyD88 at initial stages of *A. hydrophila* infection in zebrafish. TLR4 also activates TRIF signaling in endosome after relocation from the plasma membrane.^36–38^ In accordance to this, we observed MyD88 expression replaced by TRIF at later stages of infection. Our immunofluorescence results revealed TLR4-associated bacteria on the plasma membrane at 3 h, when MyD88 expression was highest and inside the early endosomes at 1 day p.i., the time point when TRIF expression was significant. TLR9 is an endosomal marker and its expression is triggered by unmethylated bacterial CpG-containing DNA motifs.^39^ We reasoned that the presence of *A. hydrophila* in endosome would trigger TLR9 expression. Elevated TLR9 mRNA expression in 1 day post-infected spleen further confirmed the presence of *A. hydrophila* inside the endosome. Our results corroborate earlier findings of Reyes-Becerril *et al*. (2011) on upregulation in TLR9 expression in spleen, intestine and headkidney of *Sparus aurata* following experimental infection with *A. hydrophila*. Based on these findings, we propose that *A. hydrophila* exploits TLR4 topology to bypass MyD88-mediated pro-inflammatory responses and survive in host.

We were interested to understand the translocation of TLR4 to endosome. It has been observed that the PI(3)K isoform p110*δ* directs translocation of TLR4 from the plasma membrane to endosome.^6^ We studied p110*δ*-mRNA expression and observed its gradual upregulation which was significant at 1 day p.i., suggesting the importance of p110*δ* in the endosomal translocation of TLR4. Our observations corroborate earlier findings in mammals,^6^ suggesting this functional attribute of p110*δ* to be evolutionarily conserved.

The next step was to study the involvement of downstream molecules IRAK4, RIP1 and TRAF6 in TLR4-associated signaling consequent to *A. hydrophila* infection. Activated MyD88 recruits members of the IRAK family,^40^ and in accordance to this we observed upregulated IRAK4 expression only at the earlier time points that coincides with increased MyD88 expression in infected spleen. This suggested activation of MyD88–IRAK4 axis, an early event in TLR4 signaling during *A. hydrophila* pathogenesis in zebrafish. RIP1, member of the serine–threonine protein kinase family related to IL-1 receptor-associated kinases, determines cellular fate following pathogen recognition.^41^ It serves as a bridge linking MyD88 and TRIF pathways through the common adaptor TRAF6. We observed significant upregulation of RIP1 at 1 day p.i. overlapping the expression of TRIF in the infected zebrafish spleen. RIP1 and IRAKs activate TRAF6, which is an adaptor shared by both MyD88 as well as TRIF.^42^ TRAF6 catalyzes the activation and translocation of freed NF*κ*B to the nucleus.^43^ We observed significant upregulation in TRAF6 at 1 and 2 days p.i., suggesting its leading role in TRIF-mediated pathway in *A. hydrophila* pathogenesis. Upregulation of IRAK4 and TRAF6 has been reported in fish against several bacteria including *A. hydrophila*.^30,44,45^

TRAF3, the other member of TRAF family, is recruited to TRIF prompting the release of type I interferon *α*/*β*.^46^ It has recently been observed that Type I interferons play key role in the regulation of immune and tissue homeostasis upon bacterial insult which may have beneficial or detrimental consequences for the host.^47^ We checked for the expression of TRAF3 and failed to get any induction of this gene, suggesting no-role of this pathway in *A. hydrophila* pathogenesis in zebrafish.

NF*κ*B is activated by both MyD88- and TRIF-dependent pathways.^48^ The activation of RIP1 is essential for the induction of NF*κ*B *via* the TLR4–TRIF axis.^49,50^ We observed significant expression of RIP1 at later stages of infection overlapping the expression of TRIF and NF*κ*B, suggesting the importance of TRIF-induced NF*κ*B activation in *A. hydrophila* pathogenesis in zebrafish. NF*κ*B promotes the production of both pro- and anti-inflammatory responses affecting the outcome of immune responses and disease prognosis.^51^ Our histological studies suggested that *A. hydrophila* did not induce profound inflammatory changes in the spleen. In line with this, qPCR results revealed that *A. hydrophila* infection led to a significant fold increase in expression of several anti-inflammatory cytokine genes. The expression of pro-inflammatory cytokine genes like TNF-*α* and IL-1*β* remained similar to that of controls, while we failed to detect the expression of IFN-*γ* and IL-12, indicating *A. hydrophila* induces robust anti-inflammatory responses in the spleen of zebrafish. Pro-inflammatory response enables the host to eliminate offending pathogens^52^ while anti-inflammatory responses promote pathogen survival inside the host.^53^ We hypothesized that the anti-inflammatory milieu helps *A. hydrophila* to persist inside zebrafish and cause pathogenesis. To test this, zebrafish were infected with *A. hydrophila* and bacterial colonization studies showed the bacteria indeed persisted in the infected fish. Our observation of increase in red pulp area in the histopathological assessment also coincides with our cytokine pattern of expression as the activation of platelets leads to the production of IL-10.^54^ Based on these observations we propose that *A. hydrophila* selectively activates the TRIF- NF*κ*B axis to produce anti-inflammatory cytokines minimizing collateral tissue damage and facilitating its survival and pathogenesis in zebrafish. Contrary to our results there are also reports on enhanced expression of pro-inflammatory cytokines in different fish tissues consequent to *A. hydrophila*.^28–30^

Our study suggested *A. hydrophila* escapes from endosome to cytoplasm of splenic macrophages 2 days p.i. The concomitant decline in TLR9 expression further supports bacterial egression from endosomes.^55^ Endosomes fuse with lysosome, facilitating the degradation of internalized bacteria. Thus endosomal escape is a ploy for pathogens to avoid lysosomal degradation and replicate in the host cytosol.^56,57^ RAB7 expression is frequented with the maturation of late endosomes that fuse with lysosomes.^58^ We observed downregulated RAB7 in 2 days post-infected spleen, suggesting *A. hydrophila* manipulates endosomal maturation to prevent endosome–lysosome fusion and use cytosol as the niche for replication and inducing pathogenesis. To the best of our knowledge, this is the first report on endosomal escape of *A. hydrophila.* How *A. hydrophila* prevents terminal endosome maturation and escapes to cytosol is not clear and we are trying to identify virulence factors involved in the process.

The presence of bacteria in cytosol is detected by cytosolic NOD-like receptors (NLR). NOD1 and NOD2 are important NLRs that recognize peptidoglycan present on Gram-negative bacteria to activate NF*κ*B-mediated pro-inflammatory responses.^59^ Although the presence of both NOD1 and NOD2 has been reported in fish, little is known about their role in regulating fish immune responses.^60^ As NOD2 is a macrophage-specific protein,^61^ we studied NOD2 expression in the spleen from 2 days p.i., which corresponds the time when the bacteria were detected in cytosol. The significant inhibition in NOD2 expression implicated *A. hydrophila* actively represses NOD2 expression from activating the pro-inflammatory machinery and survives in cytosol causing pathology.

How *A. hydrophila* survives and induce chronic pathology and fish death is not well known. We propose that *A. hydrophila* enters the host cell *via* TLR4, thereafter using receptor topology it migrates to endosome escaping pro-inflammatory responses. Therein, it induces anti-inflammatory responses, interferes with terminal endosomal maturation and escapes to cytosol. In the cytosol it represses NOD2 creating a favorable niche for survival and disease prognosis.

## Materials and methods

### Zebrafish care and maintenance

Healthy zebrafish (0.47±0.09 g) were purchased locally (Aquazona Exports, Delhi, India) and maintained in 20 l glass aquarium under natural photoperiod at controlled room temperature (25±2 °C). Fish were fed daily with earthworm fish flake food (Tetra bits) and acclimatized for 15 days before infection. Zebrafish maintenance was done according to the guidelines set forth by the Animal Ethics Committee of Government of India and University of Delhi (DU/ZOOL/IAEC-R/2013/32).

### mCherry tagging of *A. hydrophila*

*A. hydrophila* (strain no. 500297, NICED, India) was used for the study. The bacterial growth conditions and methods to confirm the pathogenicity of the strain have been described earlier.^31,32^ The integrative expression vector pBD64 containing mCherry-encoding gene was introduced into *A. hydrophila* by conjugation. Conjugation was performed between the recipient bacteria, *A. hydrophila* and the donor bacteria *E. coli* strain *β*-2163 (DAP auxotroph). Briefly, freshly grown donor and recipient cultures were mixed (1:2 ratio) thoroughly, centrifuged (3300×*g*; 2 min) and suspended in fresh Luria broth (LB) supplemented with 0.3 mM DAP. The bacterial mixture were incubated overnight at 37 °C on Luria agar plates supplemented with 0.3 mM DAP. The mating mixture were re-suspended in 2 ml of sterile LB with appropriate antibiotics, serially diluted and plated on selection plates containing arabinose (0.1%), ampicillin and zeocin (resistance for recipient and donor strains, respectively) and incubated overnight at 37 °C. The conjugants grown in the presence of both antibiotics were selected for further studies. The transconjugants were confirmed by wide-field microscopy for mCherry expression. These transconjugants did not show any significant difference in virulence (data not shown).

### Bacterial colonization

Zebrafish (10 fish per group) were injected intraperitoneally (i.p.) with different numbers of *A. hydrophila* (1×10^6^–1×10^9^ CFU in 25 *μ*l 0.6% saline). Fish mortality was recorded for 4 days and LD50 dose was calculated using the graphical method. A sublethal dose causing 7% mortality (6×10^6^ CFU) was selected for the further studies. The number of bacteria injected was determined retrospectively by plating on *Aeromonas* selective media supplemented with 100 gm/l ampicillin (HiMedia, Mumbai, India). Control zebrafish were injected with 0.6% saline.

The *A. hydrophila* isolate used in this study is sensitive to chloramphenicol and resistant to ampicillin.^31^ Zebrafish were maintained in chloramphenicol treated water for 7 days and transferred to fresh water and kept for 15 days to remove the residual antibiotics. Fish were randomly sampled, killed and plated on *Aeromonas* selective medium supplemented with ampicillin to detect the presence of residual *A. hydrophila*. The absence of bacterial colonies suggested the fish to be *A. hydrophila* free. Subsequently, these fish were infected with *A. hydrophila* and at indicated time points p.i. killed, homogenized, plated on selective media and incubated at 30 °C for 24 h for enumerating the CFU. The number of colonies were counted and expressed as Log_10_CFU/g body weight.

### Histopathology of spleen

Zebrafish uninfected and infected with *A. hydrophila* were cold anesthetized,^62^ spleen removed at indicated time points, washed and fixed in aq. Bouin’s fluid. The fishes were killed after sampling. The fixed tissues were dehydrated in an ethanol series of ascending concentrations, cleared in cedar wood oil and embedded in paraffin wax (m.p. 58–60 °C). Serial sections (6 *μ*m; Spencer microtome; Medimeas, Haryana, India) were mounted on ethanol-cleaned glass slides, kept at 37 °C overnight, de-parafinized in xylene, hydrated in a descending ethanol series and stained with Ehrlich hematoxylin and eosin. The stained sections were dehydrated in an ascending ethanol series, cleared in xylene, then mounted in DPX and viewed under a light microscope (×40, Nikon Eclipse 400; Nikon Instech Co. Ltd., Kangawa, Japan).

### RNA extraction and cDNA synthesis

Spleen was removed from control and *A. hydrophila*-infected fishes at indicated time points and total RNA extracted using TRI Reagent (Sigma-Aldrich Corp., Darmstadt, Germany). After determining the purity and concentration, the RNA samples were incubated in 1 *μ*l of reaction buffer containing MgCl_2_ (20 mM) and 1 *μ*l of RNase free DNase I at 37 °C for 30 min to eliminate genomic DNA contamination. The reaction was stopped using 1 *μ*l of EDTA (50 mM) at 65 °C for 10 min and RNA reverse transcribed using the Revert Aid First Strand cDNA Synthesis Kit (Thermo Fisher Scientific, Waltham, MA, USA). The cDNA was diluted (1:100) in nuclease-free water and stored at −20 °C for further use.

### Real-time quantitative PCR

The PCR mixture (total volume 6 *μ*l) contained 3 *μ*l of SYBR AmpliTaq Gold DNA Polymerase (ABI), 1 *μ*l of cDNA, forward and reverse primers (0.2 *μ*l each) and DEPC water (1.6 *μ*l). The primers used for qRT-PCR are listed in Table 1. The comparative Ct (ΔΔCt) method was used to evaluate the expression of candidate genes using Real-Time PCR (ABI ViiA; Applied Biosystems, Foster City, CA, USA). β-Actin was used as the internal calibrator to calculate the expression of the candidate genes. The fold change in expression was used as a relative measure of gene expression.

### Isolation of zSPM

Zebrafish were infected (i.p.) with or without mCherry-tagged *A. hydrophila* (6×10^6^ CFU), spleen removed at indicated time points and zSPM isolated. Briefly, spleens were placed in RPMI-1640 (Gibco/Life Technologies, Carlsbad, CA, USA), single-cell suspensions of total splenic cells were prepared using nylon mesh, washed and incubated for adherence in a Petri plate in a CO_2_ incubator (5% CO_2_; 4 h; 30 °C). After incubation, non-adherent cells were removed and adherent cells were collected in a 15 ml centrifuge tube using chilled RPMI-1640. Cells were then washed at 400×*g* (4 °C; 10 min) and re-suspended in 1 ml fresh RPMI-1640. The macrophages thus obtained were checked for purity by staining with Wright-Giemsa stain as well as on a flowcytometer (FSC vs SSC; BD Accuri; BD Biosciences, San Jose, CA, USA) and the viability determined using the 0.4% trypan blue dye exclusion method.

### Immunofluorescence studies

#### For bacterial trafficking

zSPM isolated from control and infected (mCherry tagged) fish were re-suspended in RPMI-1640 supplemented with 10% FBS (Gibco/Life Technologies), seeded on ethanol-cleaned glass slides and incubated for 30 min for their adherence under humid conditions. The adhered zSPM were sequentially treated with fixation buffer (BD Cytofix; 10 min), permeabilization buffer (0.1% Triton X; 15 min), washed twice with PBS and incubated with antibody for early endosomal marker EEA1 (Abcam, Cambridge, UK, 1:500) overnight at 4 °C. The cells were washed with PBS, incubated with secondary antibody (rabbit anti-mouse IgG–FITC; Santa Cruz Biotechnology Inc., Dallas, TX, USA) for 3 h at room temperature. The cells were stained with Hoechst (100 *μ*g/ml; 15 min; Sigma-Aldrich Corp.), mounted in fluoroshield (Sigma-Aldrich Corp.) and viewed under a fluorescence microscope (×100; Zeiss Imager, Z2; Carl Zeiss Iberia, SL, Madrid, Spain).

#### For TLR4 trafficking

zSPM were isolated at indicated time points and permeabilized as described above. The cells were then incubated with antibody for TLR4 (rabbit anti-TLR4, GTX113024; GeneTex, Irvine, CA, USA; 1:1000) overnight at 4 °C followed by incubation with antibody for early endosomal marker EEA1 for 4 h. The cells were washed with PBS, incubated with secondary antibody (goat anti-rabbit IgG- TRITC (Santa Cruz Biotechnology Inc.) for TLR4, 1: 250 and rabbit anti-mouse IgG–FITC for EEA1; 1:250) for 3 h at room temperature. The cells were washed, stained with Hoechst (100 *μ*g/ml; 15 min; Sigma-Aldrich Corp.), mounted in fluoroshield (Sigma-Aldrich Corp.) and viewed under a fluorescence microscope (×100; Zeiss Imager, Z2, Carl Zeiss Iberia).

### Statistical analysis

One-way ANOVA followed by Dunnett *post hoc* test was performed (SPSS software, Version 13; SPSS Inc., Chicago, IL, USA) to determine statistical significance between control and the experimental groups. Differences were considered significant when *P*<0.05.

## Additional information

**Publisher’s note:** Springer Nature remains neutral with regard to jurisdictional claims in published maps and institutional affiliations.

## Figures and Tables

**Table 1:** List of real-time primers used for indicated genes of zebrafish

*Gene*	*Forward primer*	*Reverse primer*	*Accession number*	*Product length (bp)*
*β Actin *(*actb1*)	CGAGCAGGAGATGGGAACC	CAACGGAAACGCTCATTGC	FJ915059.1	104
*TLR4a*	CGGCACTCCTCAAATCAACT	GTCCTTCAAATCCTCCCACA	NM_001131051	77
*TLR9*	CACGGACACCCAGTATGATG	GGAATCCAGTCACGCTCTTC	NM_001130594	147
*MyD88*	AGTTTGCGCTCAGTCTTTGC	ACAGATGGTCAGAAAGCGCA	NM_212814.2	110
*TRIF *(*ticam1*)	GCCGAAGAGTCGTAGACCTG	CCTCTCCCAAATTCCGTCCC	NM_001044759.1	167
*IRAK4*	TACTGGACGAGGGTTTTGTGG	CGCACTCGAGCTATCCTTCATC	BC164777.1	153
*RIP1 *(*ripk1l*)	TCCTGGACCAAACCATCAGC	ACTGACTCCTCAATTCGGGC	NM_001043350.1	100
*TRAF6*	ACTAGAGGAGAGCACCCGAG	GGAGGACAATAGGCTGACCG	NM_001044752.1	119
*TRAF3*	GCTGTGGTCTCCAAAACCCT	GAGCGAGAGATGTGTGCCTT	NM_001003513.1	126
*NFκB* *(relA*)	AAAAGATGGAGCCCTCACCC	ATCAGCCTTGCATCCCTACC	NM_001001839.2	148
*TNF-α*	GCGCTTTTCTGAATCCTACG	TGCCCAGTCTGTCTCCTTCT	AY427649	149
*IL-1β*	TGGACTTCGCAGCACAAAATG	CGTTCACTTCACGCTCTTGGATG	AY340959	151
*IFN-γ*	ATGATTGCGCAACACATGAT	ATCTTTCAGGATTCGCAGGA	AB158361	189
*IL-12*	AGCAGGACTTGTTTGCTGGT	TCCACTGCGCTGAAGTTAGA	AB183001	145
*IL-4*	CATCCAGAGTGTGAATGGGA	TTCCAGTCCCGGTATATGCT	AM403245.2	200
*IL-10*	ATTTGTGGAGGGCTTTCCTT	AGAGCTGTTGGCAGAATGGT	NM_001020785	199
*P110δ* (*pik3cd*)	TCTCTGGTCACTCGAACCGA	GTGCTTTCTCCTGACCCGAA	NM_201199.1	100
*RAB7*	ATCACTGGCCTTTGTAGACGAG	GGCGATTTTGCAGAAGTGGTG	NM_200928.1	155
*NOD2*	GAGTTCTGTTGTGACTGGGCT	TCGCTACCTCCACCACATAGA	NM_001328044.1	114
